# Acromegaly and Colorectal Neoplasm: An Update

**DOI:** 10.3389/fendo.2022.924952

**Published:** 2022-06-20

**Authors:** Leandro Kasuki, Bernardo Maia, Mônica R. Gadelha

**Affiliations:** ^1^ Endocrine Unit and Neuroendocrinology Research Center, Medical School and Hospital Universitário Clementino Fraga Filho - Universidade Federal do Rio de Janeiro, Rio de Janeiro, Brazil; ^2^ Neuroendocrine Unit - Instituto Estadual do Cérebro Paulo Niemeyer, Secretaria Estadual de Saúde, Rio de Janeiro, Brazil; ^3^ Neuropathology and Molecular Genetics Laboratory, Instituto Estadual do Cérebro Paulo Niemeyer, Secretaria Estadual de Saúde, Rio de Janeiro, Brazil

**Keywords:** acromegaly, colon cancer, colon polyp, mortality, colonoscopy

## Abstract

Acromegaly is a systemic disease caused by excessive inappropriate secretion of GH and IGF-I levels, resulting in many systemic complications, including cardiovascular, respiratory, metabolic diseases, and a possible increased risk of some neoplasias. Although many studies on acromegaly and cancer remain uncertain, most data indicate that colorectal cancer (CRC) incidence is increased in this population. The exact mechanism involved in the role of GH-IGF-I axis in CRC has not been fully explained, yet it is associated with local and circulating effects of GH and IGF-I on the colon, promoting angiogenesis, cell proliferation, risk of mutation, inhibition of tumor-suppressor genes and apoptosis, thus facilitating a tumor microenvironment. Nevertheless, population-based studies present controversial findings on CRC incidence and mortality. All worldwide guidelines and expert consensuses agree with the need for colonoscopic screening and surveillance in acromegaly, although there is no consensus regarding the best period to do this. This review aims to analyze the existing data on CRC and acromegaly, exploring its pathophysiology, epidemiological studies and their limitations, colonic polyp characteristics, overall cancer and CRC incidences and mortality, risk factors for colon cancer pathophysiology, and recommendation guideline aspects.

## Introduction

Acromegaly is a chronic systemic disease caused by the excessive secretion of growth hormone (GH) and consequently increased insulin-like growth factor type I (IGF-I) levels. In approximately 98% of cases, acromegaly is caused by a GH-secreting pituitary adenoma (somatotropinoma) (1, 2). Almost all epidemiologic studies are consistent in that acromegaly affects both sexes equally, although a Korean study showed that its incidence is slightly higher in females (1:1.3) (3–5). Nevertheless, all studies indicate that men are younger than women by 4.5 years at diagnosis, and it usually occurs at the fourth or fifth decades of life (6). Unfortunately, there is still a delay of 5-10 years between symptom initiation and diagnosis, resulting in years of morbidity and increased mortality when not properly treated (7).

Acromegaly is associated with many systemic complications secondary to untreated chronic excess GH and IGF-I, including cardiovascular and respiratory diseases, metabolic complications, bone disease (especially vertebral fractures), arthropathy, and a possible increased risk of some neoplasias (8–10). These remarkable complications reduce the health-related quality of life and life expectancy of these patients, although the effective control of GH and IGF-I excess is able to reduce the burden of the disease and the mortality rates to normal levels observed in a general population (11–14). While cardiovascular disease has been the leading cause of mortality in the past decades, recent data suggest that cancer may be the main cause of death in acromegaly (15–17).

Although the discussion of the relationship between acromegaly and cancer dates to the last century, many uncertainties still remain in this field. Knowledge on this subject is not always homogeneous, but most studies indicate that colorectal cancer (CRC) is the main neoplasm associated with acromegaly (18, 19). Nevertheless, there is no consensus in the literature on the best approach for its screening and follow-up in these patients.

In this manuscript, we review CRC in acromegaly, discussing the GH-IGF-I axis in cancer (especially in CRC), risk factors for CRC, specific characteristics of colonic polyps, the limitations of colonoscopies in this population, data from epidemiological studies and their biases, and the different guideline recommendations for CRC screening in acromegaly.

## Materials and methods

We searched the MEDLINE/PUBMED databases up to 1979 to 2022 to identify all relevant English language medical literature for studies under the search text terms; acromegaly AND (colorectal cancer OR colon cancer OR colon polyps OR colorectal polyps).

## Pathophysiology: Somatotrophic System and Colorectal Cancer

In nonacromegaly patients, most CRCs develop as a result of a multistep transformation of normal colonic epithelium to benign adenomatous colonic polyps, severe dysplasia and, finally, an invasive and/or metastatic cancer (20–22). The process involved in the tumorigenesis of sporadic forms of CRC, which takes approximately 10–15 years, requires an accumulation of genetic and epigenetic alterations in oncogenes and tumor suppressor genes (20, 22, 23).

The first step of this classical sequence is *adenomatous polyposis coli* (*APC*) gene inactivation, a “gatekeeper” gene that regulates growth by inhibiting proliferation or promoting cell death, which causes adenoma development (20, 23). This is followed by *Kirsten rat sarcoma viral oncogene homolog* (*KRAS*) activating mutation, promoting adenoma growth; loss of heterozygosity at chromosome 18q (a tumor suppressor loci), due to chromosomal instability, a condition of malfunctioning segregation of sister chromatids during mitosis, allowing adenoma progression; and inactivation of the tumor-suppressor gene *p53*, which triggers the final transition to carcinoma (20, 23, 24). All these processes are associated with other genetic mutations, microsatellite instability, and epigenetic alterations, resulting in clinicopathological tumor features (20, 23).

When assessing the role of GH in oncogenesis, it is necessary to note that in addition to the endocrinological function related to pituitary production, it is also expressed in extrapituitary tissue, exercising autocrine and paracrine functions (25). In the normal colon, GH expression is low, but in conditions predisposing to colon adenoma or adenocarcinoma, it is exuberant (25). Although GH is not expressed within epithelial tumor cells in human colon adenocarcinoma, it is expressed in fibroblasts surrounding malignant colon carcinoma (25). In contrast, the GH receptor (GHR) is expressed in both colon epithelial and stromal cells (25). Currently, it has been postulated that both circulating (endocrine pattern) and local (autocrine/paracrine pattern) high GH levels, acting through the GHR, suppress *p53*, *APC*, DNA damage repair pathways and apoptosis and stimulate epithelial-mesenchymal transition by increasing the transcription of key metastasis-related genes, including proteins such as matrix metalloproteinases, cMYC (master regulator of cell cycle entry and proliferative metabolism), BCL-2 (B-cell lymphoma 2), and CHOP (C/EBP homologous protein 10), allowing a change in the normal intestinal mucosal environment in favor of a tumor microenvironment toward cell motility and invasion (25–27).

Another pathway that has been explored is that peroxisome proliferator-activated receptor gamma (PPARᵧ), a member of the nuclear hormone receptor superfamily that plays an important role in adipocyte differentiation and metabolism, also has an antiproliferative effect in several tissues, including colonic mucosa, where it is highly expressed (28). Although controversial, *in vitro* and animal studies suggests that PPARᵧ has an antitumor effect in CRC as its activation is associated with inhibition of cell growth and its intestinal deficiency is associated with enhanced tumorigenicity in mice small intestine and colon (28). The molecular mechanism for the antineoplastic effect of PPARᵧ activation remains incompletely enlightened. PPARᵧ ligand treatment is associated with gene expressions changes involving: induction of apoptosis, by upregulating the proapoptotic protein BAX and downregulating the antiapoptotic BCL-2; cell proliferation inhibition through the decrease in cyclin D1 expression, a downstream effector of diverse proliferative and transforming signaling pathways, leading to the arrest of cell cycle progression; induction of cellular differentiation; and inhibition of angiogenesis in CRC, by decreasing vascular endothelial growth factor (VEGF) production and inhibiting capillary endothelial cell proliferation (28). Genetic studies showed that the presence of somatic loss-of-function mutations in the gene encoding PPARᵧ contributes to colonic tumor development (29). Although some studies failed to detect any mutation of *PPAR*ᵧ of colonic tissue in patients with acromegaly, others have observed that patients with active, untreated acromegaly had lower levels of PPARᵧ expression in colonic mucosa than those with cured disease (30, 31). There is still a need for further studies, but this might have the same role of the somatic mutations in PPARᵧ, playing a role in the development and/or progression of these cancers in nonacromegaly patients (29).

In addition to the GH-IGF-I axis being able to favor tumor development, it increases the risk of mutations, stimulates cell proliferation and angiogenesis (IGF-I is expressed in endothelial cells during angiogenesis and increases vascular endothelial growth factor, the main proangiogenic factor responsible for neovascularization), invasion, and metastasis; other components of the somatotropic system, such as IGF-II and IGF binding proteins (IGFBPs), exert an antitumoral effect by stimulating apoptosis and inhibiting mitogenesis, although the strength of these actions are weaker (27, 32–35).

**Figure 1:**
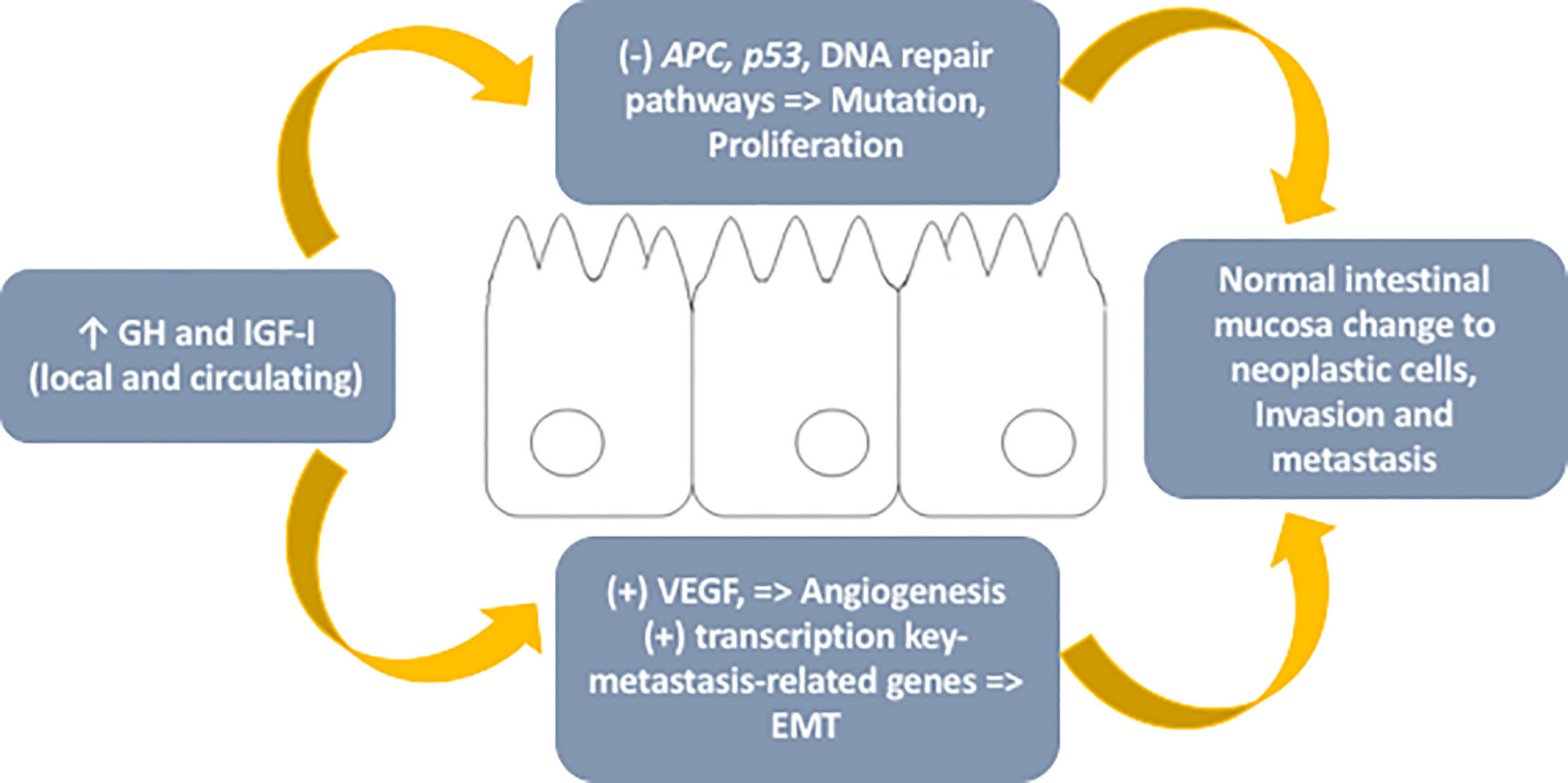
– Schematic representation of mechanisms involved in the role of the GH-IGF-I axis in colorectal cancer. GH, growth hormone; IGF-I, insulin-like growth factor type I; APC, adenomatous polyposis coli gene; VEGF, vascular endothelial growth factor; EMT, epithelial-mesenchymal transition.

The final action of this axis is complex and not yet fully understood, although the data indicate that there is an imbalance in favor of neoplastic development (**Figure 1**).

## Risk Factors for Colon Cancer Pathophysiology

The transformation of premalignant to malignant lesions, responsible for the development of CRC, corresponds to a complex process that occurs over years and is influenced by numerous factors (20, 36–38). Countless evidence points out that among these predisposing factors are diet, obesity, diabetes mellitus, dyslipidemia, physical inactivity, smoking, alcoholism, and genetic factors (36–38).

Among these factors, obesity, diabetes mellitus, and hypertriglyceridemia represent the ones with the most robust evidence, reflecting the main role of insulin resistance and hyperinsulinemia in association with its inflammatory markers in the carcinogenesis of the nonacromegaly population (37, 38). Although it is still debated, evidence points out that metformin has a protective role in patients with diabetes and insulin resistance as an anticancer chemoprevention agent (39, 40). The underlying mechanism of metformin antitumoral activity is not fully understood, but its action in reducing insulin levels and inhibiting phosphorylation of IGF-I receptor/insulin receptor and its pathways inhibition of cell proliferation and growth, as well as inducing apoptosis, have an important role (41, 42). Insulin promotes tumorigenesis by direct and indirect mechanisms, including one referring to the increase in circulating levels of IGF-I and IGF-II, through the reduction of IGFBPs (43). In addition, it is known that IGF-I, IGF-II, and insulin can cross-bind to each other’s receptors with a lower affinity than with their original receptor due to the homology presented between them (34). The similarity between IGF-I receptor (IGF-IR) and insulin receptor (IR) allows the formation of hybrid receptors, which have a higher affinity for IGF-I than insulin (34). The biological significance of this is not entirely known, but it may be implicated in a greater activation of the IGF-IR pathway and its consequent mitogenic activity.

In CRC, evidence points out that activation of the IR pathway by insulin promotes cell growth and proliferation and that activation of the same signaling route (IR) by IGFs contributes to the oncogenic process by decreasing apoptosis and stimulating angiogenesis, cell proliferation and migration (44, 45). These comorbidities are very often present in acromegaly, being related to disease activity and/or its treatment, and might contribute to an increased risk of CRC.

## Overall Cancer And Colorectal Cancer and Polyp Incidence and Mortality

In the past 20 years, data from laboratory, animal, and human model studies indicated that GH and IGF-I are associated with cancer, although findings from population-based studies present controversial results, explained by the numerous biases that will be mentioned ahead in this review (46, 47).

A review of six nationwide cohort studies that included a standardized incidence ratio (SIR) comparing the overall cancer rate and the CRC rate in patients with acromegaly with the general population indicated that five studies pointed to an increased risk of neoplasia, albeit moderate and not always with statistical significance (**Table 1**) (18, 19, 48–51). This may explain why previous smaller studies (of less statistical power) present such incongruent results. Only one of these studies showed that CRC incidence was nonsignificantly lower than expected in the general population (48). This controversial result might be explained by some limitations of the work: only a small sample of the German Acromegaly Registry was used; part of cancer data was obtained by phone interviews and not always based on medical records; and 16% of patients were lost during follow-up.

**Table 1:** Overall cancer and colorectal cancer incidence in acromegaly in nationwide studies.

Country	Patients (N)	Follow-up (years or person-years)	Overall Cancer (N)	SIR overall cancer (95% CI)	*P* value(overall cancer incidence x general population)	Colorectal Cancer (N)	SIR colorectal cancer (95% CI)	*P* value (colon cancer incidence x general population)
**Denmark, 2018** ^(18)^	**529**	**13.6**	**81 (15.3%)**	**1.1 (0.9-1.4)**	**NA**	**10 (12.3%)**	**1.4 (0.7-2.6)**	**NA**
**Italy, 2017** ^(19)^	**1512**	**10**	**124 (8.2%)**	**1.41 (1.18-1.68)**	**<0.001**	**20 (16.1%)**	**1.67 (1.1-2.6)**	**0.022**
**Germany, 2015** ^(48)^	**445**	**6656**	**46 (10.3%)**	**0.75 (0.55-1.0)**	**0.051**	**4 (8.7%)**	**0.61 (0.2-1.6) (p=0.43)**	**0.61**
**Finland, 2010** ^(49)^	**331**	**10.7**	**48 (14.5%)**	**1.5 (1.1-1.9)**	**NA**	**6 (12.5%)**	**1.9 (0.7-4.1)**	**NA**
**Sweden and Denmark, 2002** ^(50)^	**1634**	**Sweden = 10.3** **Denmark = 9**	**177 (10.8%)**	**1.5 (1.3-1.8)**	**NA**	**23 (13.0%)^a^** **13 (7.3%)^b^**	**2.6 (1.6-3.8)^a^** **2.5 (1.3-4.2)^b^**	**NA**
**United Kingdom, 1998** ^(51)^	**1239**	**16778**	**79 (6.37%)**	**0.76 (0.60-0.95)**	**1**	**12 (15.2%)^a^** **4 (5.1%)^b^**	**1.68 (0.9-2.9)^a^** **0.86 (0.2-2.2)^b^**	**0.06^a^** **0.69^b^**

SIR, standardized incidence ratio; NA, not available; ^a^, colon cancer; ^b^, rectum cancer.

Therefore, similar to overall cancer, the SIR of CRC compared to the general population has been variable, but most studies indicate an increased risk in acromegaly (18, 19, 49–51). This finding reflects the consensus of an active search for this comorbidity, although there is no uniform recommendation on how to perform this surveillance (8, 10, 13, 52–54).

Mortality in acromegaly has traditionally been related to its cardiovascular and respiratory complications (11, 51). However, current data point to a change in this paradigm, where neoplasia assumes the role of the main cause of mortality (15–17). This finding is accompanied by a general decrease in mortality when compared with older previous studies, reflecting the treatment progress made in recent years (16, 55). Thus, the decrease in overall mortality, the increase in life expectancy, and the aging of this population resulted in a consequent expected increase in the incidence of relevant age-dependent diseases, such as CRC.

Although the overall mortality rate, as well as cancer-related mortality, in controlled acromegaly are similar to that of the general population, those individuals with active disease and persistently high levels of GH and IGF-I will have higher all-cause mortality rates (between 1.5- and 2.0-fold), including those related to neoplasms, particularly CRC (15, 51, 56).

Although data related to CRC remain a subject of discussion, the increased risk for colon polyposis (either adenomatous or hyperplastic) in acromegaly is widely accepted (9). In addition, there is also a higher prevalence of diverticular disease, hemorrhoids, and the typical enlarged colon length (dolichocolon) (57).

Disease activity is directly related to the appearance of new polyps (9, 47). Excessive IGF-I levels, but not GH, and the duration of disease activity seem to correlate positively with the development of polyps in several studies (57, 58). However, there appears to be no relationship between IGF-I levels and polyp size (57).

These adenomatous polyps have some particular characteristics because, in general, they are larger, multiple, and more dysplastic than in the nonacromegaly population (59). They are also more common in men, in patients with an active disease duration greater than five years, in those with three or more skin tags, and in cases with a positive family history of colonic polyps (47, 60).

## Limitations of Epidemiological Studies and of Colonoscopy in Acromegaly

The real relationship between acromegaly and cancer remains an unsolved question (46, 47, 61). Several data have reported that GH-IGF-I axis contributes to an important role in cancer development and progression, although the excess risk seems moderate (26, 33, 46, 47, 62).

Nevertheless, the studies that associate acromegaly and cancer have controversial results, often explained by the use of different epidemiological methods that are not comparable (case–control and population-based design); the retrospective nature of these studies, especially when considering that some studies date back to an era when treatment of acromegaly was less successful, so that patients with uncontrolled disease may have died by cardiovascular morbidity, for example, before entering the age when cancer is diagnosed; or the lack of an appropriate and comparable control population, which may not adjust results for confounding factors, such as sex, age, and environmental factors (46, 47, 61, 63, 64). Another limiting factor is that many studies exclude the registration of cancer before the diagnosis of acromegaly, and as the diagnosis of this disease occurs many years after the real onset, it could have a negative impact on the actual estimate of neoplasm (47, 51). In addition, as acromegaly is a rare disease, only nationwide surveys may have the statistical power to demonstrate (or not) an excess risk of cancer (61, 64).

Another important confusing bias is related to the intensity of screening. This link between excess GH and IGF-I and the risk of cancer in some studies led to the recommendation of routine screening for neoplastic pathologies, including colorectal neoplasms, which influences the reported incidence rates (18, 26).

In addition to these study limitations, there are specific technical difficulties in assessing CRC in patients with acromegaly. In contrast to the general population, 25–40% of adenomatous polyps and 50% of adenocarcinomas in acromegaly are located in the ascending and transverse colon, so it is necessary to perform a total colonoscopy instead of a simple sigmoidoscopy (64–68). Another problem that can compromise the success of the exam is the difficulty in preparing the patient, since intestinal transit in acromegaly is slower than normal subjects. This can be explained by autonomic intestinal impairment due to vagal hypertonia, a hormonal imbalance influenced by the interactions between GH and ghrelin and the action of the increased IGF-I levels, which may stimulate the proliferation of intestinal epithelial cells (69). Gut motility disturbance can also occur in treated patients with somatostatin receptor ligands (69). Therefore, standard bowel preparations could lead to suboptimal results (70). Another major problem is that colon length and circumference are often increased (dolichocolon with megacolon), making complete intubation and identification of minor lesions more difficult (70, 71). Furthermore, there are some potentially harmful limitations inherent to this invasive procedure itself that may be enhanced in acromegaly patients, such as polypectomy bleeding (more common in the proximal colon), perforation (increased in the presence of diverticular disease – increased in acromegaly), and cardiopulmonary complications (cardiac arrythmias, hypotension, oxygen desaturation) (72).

All these considerations and technical complexity suggest the need for a trained and high-level skill endoscopist to perform this exam in patients with acromegaly.

## Guidelines

There are numerous guidelines for the management of acromegaly patients, and practically all address the neoplasia risk. In relation to breast, prostate, lung, and other cancers, no increase in risk has been conclusively reported, and there is an agreement among all experts that surveillance should follow the same recommendations as for the general population (8, 9, 13, 47, 54). Specifically, with regard to the thyroid, current data do not support routine screening for thyroid cancer in acromegaly (13). Consistent with international guidelines, thyroid ultrasound is recommended in patients with clinically palpable nodules, and investigation with fine-needle aspiration cytology must respect the indications for the general population (8, 13, 54, 73).

However, in relation to CRC, there is no consensus regarding the best period for colonoscopic screening and surveillance during the follow-up of these patients. The most referenced guidelines on this topic in the literature are those published by the British Society of Gastroenterology (BSG) in 2010 (53); the American Association of Clinical Endocrinologists (AACE) in 2011 (54); the Pituitary Society in 2013 (10); the Endocrine Society in 2014 (13); and the Acromegaly Consensus Group (ACG) in 2019 (8).

According to the BSG, colonoscopy should begin at the age of 40 (52, 53). The AACE, Pituitary Society, Endocrine Society and ACG recommend that the first exam should be requested at the time of diagnosis, independent of the patient’s age (8, 10, 13, 54). If a patient has normal initial colonoscopy and normal IGF-I levels, all societies recommend a new exam every 10 years. Nevertheless, there is no agreement on the best interval if the initial or any subsequent colonoscopy reveals an adenoma and/or if IGF-I is uncontrolled. The AACE and Endocrine Society recommend surveillance every 5 years; BSG suggests 3-year colonoscopy; the Pituitary Society advises performing the exam more frequently if IGF-I remains persistently elevated (without specifying the precising time) and proposes to follow in accordance with clinical guidelines for the general population if colonoscopy is abnormal (8, 10, 13, 52–54). It is worth noting that only the Pituitary Society cite a positive family history for colorectal cancer as a risk-modifying agent in acromegaly (8, 10). A summary of current guideline recommendations for surveillance colonoscopy in acromegaly patients is presented in **Table 2**.

**Table 2:** Current guideline recommendations for surveillance colonoscopy in acromegaly.

	BSG, 2010 (53)	AACE, 2011 (54)	Pituitary, 2013 (10)	Endocrine Society, 2014 (13)	ACG, 2019 (8)
**Age at initial colonoscopy**	**≥40 years**	**At the time of diagnosis**	**At the time of diagnosis**	**At the time of diagnosis**	**At the time of diagnosis**
**Normal initial colonoscopy and normal IGF-I**	**Every 10 years**	**Every 10 years**	**Every 10 years**	**Every 10 years**	**-**
**Adenoma in the colonoscopy and/or elevated IGF-I**	**Every 3 years**	**Every 5 years**	**“More frequently”**	**Every 5 years**	**-**

BSG, British Society of Gastroenterology; AACE, American Association of Clinical Endocrinologists; ACG, Acromegaly Consensus Group.

Special attention should be given to the degree of recommendation when assessing different opinions provided by each society, since none establishes a strong or high-quality rate of evidence. For example, the BSG expresses a degree of recommendation B, characterized by evidence obtained from at least one well-designed controlled study without randomization, evidence obtained from at least one other type of well-designed quasi-experimental study, or evidence obtained from a well-designed nonexperimental descriptive study, such as comparative studies, correlation studies, and case studies (53). The AACE presents as a weak level of evidence (Grade C) based on expert opinion and on data from experimental results and nonexperimental data (54). The Pituitary Society followed the approach recommended by the Grading of Recommendation, Assessment, Development, and Evaluation (GRADE) group (74), and it has a strong degree of recommendation for the age of onset of colonoscopy and the exam time interval if the IGF-I level persists elevated (10), but it has a discretionary recommendation if the initial colonoscopy is normal and IGF-I level is normal (10). The Endocrine Society presents its colonoscopic guideline as a weak degree of recommendation with low-quality evidence studies (13). The ACG uses the same GRADE system and has a discretionary recommendation (8).

Due to the lack of conclusive data on the real incidence of malignancy and the relationship between CRC mortality and acromegaly, there exists heterogeneity in the current surveillance recommendations for this neoplasm.

Current studies point to a higher incidence of CRC in increasingly younger nonacromegaly individuals, but screening for the general population still starts from the age of 50, as indicated by gastroenterology, oncology, and endoscopy societies (21, 36). The same guidelines recommend a more intense and earlier screening and follow-up only for patients allocated as the group at major risk for CRC, since this measure has been shown to reduce the risk of specific death for this neoplasm in this population (21, 75–79). For these individuals at increased risk, such as first-degree relatives of individuals diagnosed with CRC at young ages, the beginning of screening at younger ages is recommended (starting at age 40 years or 10 years before the youngest case in the family). For high-risk groups (familial adenomatous polyposis, hereditary nonpolyposis colon cancer, or inflammatory bowel disease), much more rigorous prevention programs are recommended, starting earlier in life (21, 75, 77, 78). It is important to note that none of these specific guidelines for CRC cite acromegaly as a screening modifying disease.

It is widely accepted that screening and follow-up colonoscopy are fundamental in acromegaly. However, the correct time for the first colonoscopy and for follow-up exams remains uncertain. There is little evidence to justify colonoscopy at the time of diagnosis in patients under the age 40, since in average-risk individuals (no prior diagnosis of CRC, adenomatous polyps, or inflammatory bowel disease; no personal diagnosis or family history of known genetic disorder that predisposes them to a high lifetime risk of CRC, such as Lynch syndrome or familial adenomatous polyposis), this exam starts at 50 years (21, 47, 80).

## Conclusion

Although there are many biases and limitations in quantifying the overall CRC risk in the literature, current evidence suggests that acromegaly should be included in the group of factors associated with CRC, such as smoking, alcoholism, obesity, physical inactivity, diabetes mellitus, and others, which increase the risk for this neoplasm to a mild and moderate level (21, 36, 81).

Further studies on the topic are urgently needed for the adoption of a universal evidence-based guideline. In the near future, with more studies and data on this subject, the age of initial CRC screening in acromegaly and the interval between exams could possible be reviewed.

## Author Contributions

LK and BM reviewed the literature and wrote the manuscript. MG reviewed the manuscript and suggested the final changes. All authors contributed to the article and approved the submitted version.

## Conflict of Interest

The authors declare that the research was conducted in the absence of any commercial or financial relationships that could be construed as a potential conflict of interest.

## Publisher’s Note

All claims expressed in this article are solely those of the authors and do not necessarily represent those of their affiliated organizations, or those of the publisher, the editors and the reviewers. Any product that may be evaluated in this article, or claim that may be made by its manufacturer, is not guaranteed or endorsed by the publisher.
